# Construction Formula of Biological Age Using the Principal Component Analysis

**DOI:** 10.1155/2016/4697017

**Published:** 2016-12-06

**Authors:** Linpei Jia, Weiguang Zhang, Rufu Jia, Hongliang Zhang, Xiangmei Chen

**Affiliations:** ^1^Department of Nephrology, The Second Hospital of Jilin University, Ziqiang Street, No. 218, Changchun, Jilin 130041, China; ^2^Department of Nephrology, Chinese People's Liberation Army General Hospital, Chinese People's Liberation Army Institute of Nephrology, State Key Laboratory of Kidney Disease, National Clinic Research Center for Kidney Diseases, Fuxing Road, No. 28, Beijing 100853, China; ^3^Central Hospital of Cangzhou, Xinhua Middle Street, No. 201, Cangzhou, Hebei 061001, China; ^4^Department of Life Sciences, The National Natural Science Foundation of China, Shuangqing Road, No. 83, Beijing 100085, China

## Abstract

The biological age (BA) equation is a prediction model that utilizes an algorithm to combine various biological markers of ageing. Different from traditional concepts, the BA equation does not emphasize the importance of a golden index but focuses on using indices of vital organs to represent the senescence of whole body. This model has been used to assess the ageing process in a more precise way and may predict possible diseases better as compared with the chronological age (CA). The principal component analysis (PCA) is applied as one of the common and frequently used methods in the construction of the BA formula. Compared with other methods, PCA has its own study procedures and features. Herein we summarize the up-to-date knowledge about the BA formula construction and discuss the influential factors, so as to give an overview of BA estimate by PCA, including composition of samples, choices of test items, and selection of ageing biomarkers. We also discussed the advantages and disadvantages of PCA with reference to the construction mechanism, accuracy, and practicability of several common methods in the construction of the BA formula.

## 1. Introduction

Societies are currently facing a dramatic demographic change with an ever-increasing life expectancy, as well as an aged trend of population [1]. By 2050, the world population aged 80 and above will be more than triple, approaching 400 million individuals [2]. It is therefore urgent to plan a health administration to deal with or even prevent the senescence. Thus various studies aim at shedding light upon the mechanism of ageing and improving the life quality of the elderly [3–5]. These investigations have improved the search for ageing biomarkers to find ideal indices for evaluating or even predicting the ageing process. However, until now there has been no golden index for clinical or basic researches. Coasta and McCrae have pointed out that physical, physiological, or biochemical parameters alone or multivariate composition can only offer limited information regarding functional age in comparison to the chronological age (CA) [6].

To better evaluate the ageing process and predict the occurrence of ageing related diseases, the conception of biological age (BA) was proposed to describe the changes of body function with the same or similar CA in an objective way. The biological ageing is defined as a process or a group of processes that result in the progressive decrement of viability of the organism with advancing age [7]. Aiming at evaluating BA precisely, a kind of model was derived to construct a BA formula combining a series of ageing biomarkers with statistical method. This kind of BA formula aims at assessing the functional status during ageing as well as predicting ageing related diseases. Statistical methods that have been used for BA formula construction include the multiple linear regression (MLR), the principal component analysis (PCA), Hochschild's method, and the Klemera and Doubal method (KDM) [7–15]. Although some new methods other than PCA have been proposed and tested [8, 16], researchers still use PCA as a stable and common statistical method in some studies [12]. For example, PCA is better than MLR in avoiding some statistical deficiencies [7, 17, 18].

Here, we searched studies on the construction of the BA formula via the PCA method, including both cross-sectional and longitudinal studies. Then we further summarized the basic process and the influential factors of constructing BA equation with PCA.

## 2. Criteria of Subject Enrollment

In previous studies, subject enrollment usually followed a series of standards (Table 1). First, all the subjects are healthy or almost healthy to exclude the impacts of diseases. Thus, the constructed formula can represent the normal ageing process or even works as a disease prediction model. Some researchers excluded all the unhealthy individuals and selected the healthy ones only [17–20, 23–25]. Volunteers who had been diagnosed with hypertension, diabetes, trauma, renal failure, mental disorder, stroke, cancer, heart failure, coronary heart disease, peripheral artery disease, pulmonary diseases, or a disease history within the last 6 months were excluded [17–20, 23–25]. However, since the senescence does not have an established cut-point with some diseases and subclinical conditions, the selections of subjects in some studies were not strictly performed. Some volunteers with hypertension, dyslipidemia, diabetes, and early impairment of organs were also included [7, 11, 21, 22]. Second, researchers need to control the size and constitution of samples. In early exploratory studies, the BA formulae of males and females were analyzed separately, and the sample size was usually small, which was often less than 100 people in most circumstances [7, 17, 18, 20, 21]. Then with the development of BA formula construction, the sample size was expanded accordingly [11, 12, 19, 23–25]. Meanwhile, both males and females were comparably included, and the ratio of two genders was near to 1 : 1 in many studies, which may avoid the sexual factors caused by the gender differences of ageing process [11, 12, 22–25]. The expanding sample size and balanced ratio of two genders are required to give deeper view of BA in the whole population. Third, volunteers aged over 18 years participated so as to cover all the age groups of adults, including youth, the middle-aged, and the elderly. Ages distribute as evenly as possible to eliminate such bias. However, some researchers claimed that the age range should be from 30 to 80 by arguing that major organ functions begin to decline after 30 years and the people over 80 have their specific biological states that are different from younger people [23]. Though the age range of 30 to 80 years is a rigorous consideration, the exclusion of people aged younger than 30 or over 80 needs to be further discussed. Then the data of selected subjects were collected and analyzed to construct the BA equation.

## 3. Candidate Test Items and Ageing Biomarkers

### 3.1. Candidate Test Items

Ageing biomarkers were selected from tens or hundreds of test items (Table 2). The proper test items can reflect the ageing process of vital organs. The selection of test items is the foundation of BA formula construction. Some of the test items are routine clinical examinations, which help researchers to have a general understanding of the study subjects, such as age, gender, height, body fat, blood pressure, blood routine, blood chemistry, urine routine, and pulmonary function [7, 11, 12, 17–25]. The routine examinations were included in nearly all of the studies about BA model construction. Beyond that, some other test items were used to explore better ageing biomarkers. Some researchers added cardiovascular examinations, including cardiac ultrasound, carotid artery ultrasound, and electrocardiograph [7, 12, 23–25]. Studies have validated that some parameters of cardiovascular examinations are good biomarkers in estimation of BA [12]. Since the elderly people have a higher risk of cardiovascular system disease, and the ageing process of cardiovascular system is typical [24]. The imaging examinations were also preferred by a small number of researchers, such as abdomen ultrasound, chest radiography, and gastrointestinal endoscopy [7, 12, 23]. Due to the relatively high cost, fuzzy evaluation standards, and radioaction, the use of imaging examinations in BA estimation is largely restricted. Ageing is not a single process and may be affected by various factors such as the environment, living habits, and heredity. In order to explore how the living habits affect the ageing, questionnaires were added to record the living habits of subjects [7, 12, 21, 24, 25]. In previous studies, many factors, including smoking status and exercise levels, were shown to have a close relationship with individual ageing [21]. The advanced technology of genetics prompted people to investigate whether genetic parameters can be selected as ageing biomarkers. Telomere restriction fragment (TRF) length is recognized as a genetic marker of ageing at the cellular level, which is associated with dynamic ageing [29–31]. Zhang et al. found that the TRF length has a positive correlation with CA and is closely related to ageing biomarkers [25]. When it comes to hormones, Bai's team selected estrogen (ESTR) as one of the test items, and they reported that ESTR was a significant predictor of BA in women only [12]. It is worth thinking that more sex hormones should be tested for providing a new way to estimate BA in different genders.

### 3.2. Ageing Biomarkers

Biomarkers of ageing are the composition of BA formula. It is important in the process of construction to select the proper biomarkers, because the more accurate the biomarkers are selected, the more precise the BA formula is. Generally, biomarkers of ageing come from the vital organs that are sensitive to body ageing. Common biomarkers are summarized in Table 3. Among all the biomarkers, systolic blood pressure (SBP) has a relatively higher frequency, followed by blood urea nitrogen (BUN) and forced expiratory volume in 1 s (FEV1). SBP is known as a major marker of cardiovascular system closely associated with cardiovascular diseases. It appears that SBP increases steadily with ageing [32]. FEV1 represents the function of respiratory system. Some studies have reported that FEV1 is related to senescence and mortality [33]. Interestingly, two groups of researchers found that FEV1 was also associated with a substantial excess risk of cardiovascular diseases [34, 35]. Thus FEV1 could be a good biomarker of ageing. BUN is a traditional marker of renal function, while Cystatin C is a relatively new and more sensitive marker [36]. Cystatin C has a higher correlation with CA as compared with BUN [12, 24, 25]. Thus Cystatin C may replace BUN as the new biomarker in BA formula construction.

Early studies focused on the indices of blood chemistry and blood routine, while the latter studies added some new test items, such as cardiovascular ultrasonography, nervous system tests, IL-6, and TRF length. Among them, indices of cardiovascular ultrasonography, TRF length, and nervous system are more sensitive with body ageing [12, 24, 25]. These test items push forward the research progress. It is worth mentioning that TRF length, a genetic parameter, breaks a new path in construction of BA model. Zhang et al. have found TRF length as a benchmark to select biomarkers of ageing to build BA equation instead of CA, since TRF length has a positive correlation with CA and is closely related to other ageing biomarkers [25]. Application of TRF length indicates that other genetic markers, such as single nucleotide polymorphism (SNP), methylation, and copy number variation (CNV), may also be new benchmarks to select biomarkers of ageing [25, 31].

In many studies, parameters of respiratory system have the relatively higher coefficient with CA, especially FEV1 and forced vital capacity (FVC) [7, 11, 17, 20, 22, 23]. The *r* values of these two indices are always greater than 0.50, which shows a close relation between BA and respiratory system. Because other indices of respiratory system are not often included, a further study needs to be done. Moreover, SBP, pulse pressure (PP), mitral valve annulus lateral wall of peak velocity of early filling (MVEL), and mitral valve annulus inferior wall of peak velocity of early filling (MVEI) are found to have higher coefficients with CA, respectively [12, 18, 24, 25]. Both MVEL and MVEI are relatively new biomarkers of ageing, and the accuracy needs to be verified in the future.

## 4. Basic Steps of Construction BA Formula Using PCA

**Figure 1:**
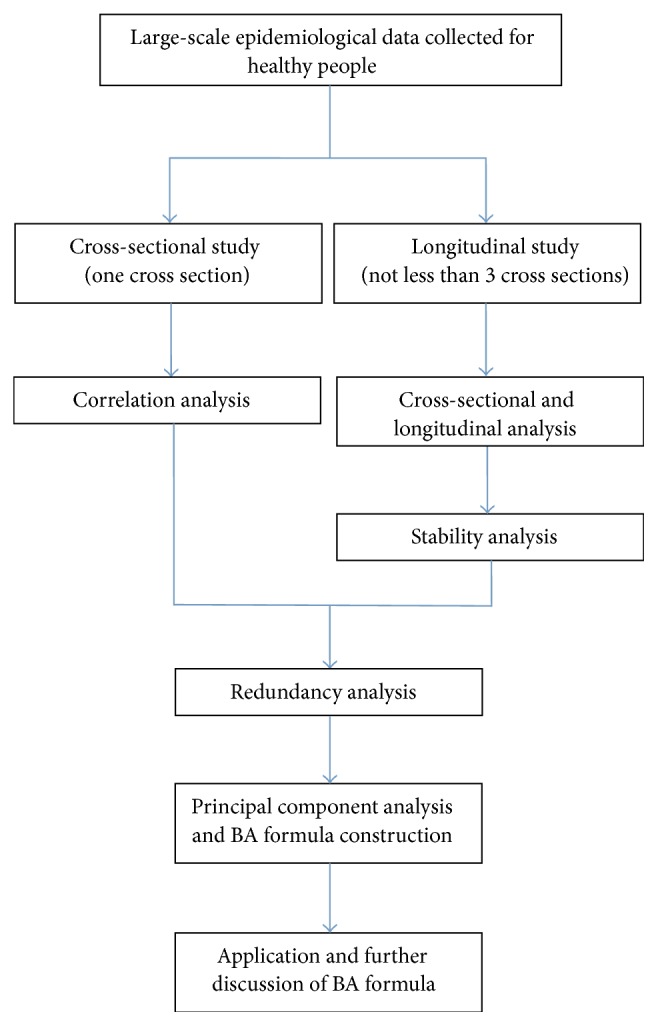
Flow chart of basic steps of biological age model constructions by principal component analysis.

The method of building BA equation using PCA usually consists of five steps: correlation analysis, stability analysis, redundancy analysis, PCA, and the equation construction (Figure 1).

Because candidate biomarkers of ageing are expected to show evidence of changes with passage of time, correlation analysis is the first step to exclude the indices that have low coefficients of correlation with CA. By correlation analysis, some of the indices, which have high relation with CA and reflect the ageing process of body, are selected as candidates of ageing biomarkers for further analyzing.

The second step is stability analysis, which only exists in longitudinal analysis. Stability analysis is used to examine the degree of longitudinal stability of individual differences to evaluate the interyear reliability of annual values of each index [20]. Considering the results of correlation analysis, indices with a higher correlation with CA and keeping stable in succeeding years are selected.

Some of the indices selected by correlation analyses and stability analyses come from one vital organ and evaluate the same part of body function; for example, both pressure pulse and SBP reflect the conditions of the vascular system. Thus redundancy analysis is the third step to eliminate the repeated indices. In redundancy analysis, correlations of each of the two indices are calculated, and the pair of indices with high coefficient is defined as repeated indices. The one with higher correlation with CA is selected.

Then PCA is applied to reduce the dimension of the variables. Indices with eigenvalues greater than 1.0 are determined as principal components, and the greater one is called the first principal component. Other variables load onto the first principal component to explain the variation of BA. Two kinds of PCA are done, with CA and without CA. PCA with CA determines the relationship between CA and principal components. While PCA without CA is used to show whether the relationships will be held without the influence of CA. Hence the biomarkers of ageing are selected.

In the last step of formula construction, the formula of biological age score (BAS) = *a∗*(*X*_1_ − mean_1_)/SD_1_ + *b∗*(*X*_2_ − mean_2_)/SD_2_ + *c∗X*_3_ + ⋯*n∗*(*X*_*n*_ − mean_*n*_)/SD_*n*_. Here *n* is the coefficient score, *X*_*n*_ is the biomarker, mean_n_ is the mean value of *X*_*n*_, and SD_*n*_ is the standard deviation of *X*_*n*_. Some researches would like to transform BAS to BA, because the unit of BAS is not year. The following method is applied to transform BAS to BA: BA = BAS (the standard deviation of CA) + the mean of CA. Because the BA formula is underestimated of the means of BA for the upper end of regression and overestimated for the lower end, researchers correct the formula using the following: corrected BA = BA +* Z*.* Z* means (*y*_*i*_ − *y*)(1 − *b*). *y*_*i*_ is the individual's CA and *y* is the average of CA.* b* stands for the coefficient of linear regression between CA and BA [7]. Here the formula of BA is constructed.

## 5. Statistical Points in BA Formula Construction Using PCA

### 5.1. Correlation Coefficient and Redundancy Coefficient

Correlation coefficient and redundancy coefficient are two main parameters in correlation analysis and redundancy analysis. They are the criteria of selection of ageing biomarkers and decided by the quality of indices. Wedam has interpreted *r* as follows: *r* > 0.7 means a strong correlation; 0.5 < *r* < 0.7 means moderate correlation; 0.3 < *r* < 0.5 means weak to moderate correlation;* r* < 0.3 means weak correlation [37]. The *P* value only indicated whether the test of *r* = 0 is right; herein the stress should be put on the value of* r* [37].

The coefficients of different studies are not unified and range from 0.12 to 0.40 (Table 4). Disparities of coefficients may arise from the selection of test items and the research requirements. Because some of the examinations better reflect the senescence of vital organs, the coefficient would be higher if these examinations are included. In the early studies, the correlation coefficients were relatively low, because the test items were mainly focused on the common examinations in clinical practice, such as blood routine, blood chemistry, and urine routine [17, 20, 23]. With the exploration of new ageing biomarkers, new test items are added into the researches, such as cardiovascular ultrasound, carotid artery ultrasound, and some inflammatory cytokines [12, 24]. New test items improve the relevance with chronological age as well as the correlation coefficient.

Coefficient of redundancy analysis is usually more than 0.6 (Table 4). However, the coefficient is not fixed, since the selection of indices is not based on the statistical analysis only, but also considered of clinical factors and requirement of the research. Certainly, a higher coefficient means better quality of data.

### 5.2. PCA versus Other Methods

Common methods of BA estimates include MLR, PCA, Hochschild's method, and KDM. MLR of CA has long been used for the calculation of BA, whereas multiple regression of equation overestimates individual BA for young people and underestimates those for the elderly [23]. Some researchers found that the distortion of the BA at the regression edge is influenced by mathematical factors of MLR, and this method also ignores the discontinuity of the ageing rate during the whole life of individuals [9, 27, 28]. Because in PCA method we use the fewer uncorrelated variables to explain the most variance, instead of combining different variables into a regression equation, PCA avoids some of the statistical deficiencies of MLR [28]. Though PCA has some advantages compared to MLR to some extent, it may not be the best formula of BA estimation. Some new methods such as the KDM and Hochschild's method have been tested and appeared better than PCA [26]. Specifically, Levine compared BA estimation constructed by MLR, PCA, and the KDM in a longitudinal study and found that the KDM was the most reliable predictor of mortality which performed significantly better than the other two methods [16]. However, the outstanding KDM seems to be complicated in computation [26]. Comparisons among MLR, PCA, Hochschild's method, and KDM are shown in Table 5.

## 6. Discipline of Ageing

### 6.1. Why Do Women Have a Longer Lifespan Than Men?

Usually women have a longer life span than men. To explore the potential causes of such a phenomenon, the BA formula constructed by PCA is widely used in discussions of sexual differences. By using BA formula, some researchers found that although women have a lower biological vigor than men in early adulthood, the ageing rate of women is up to 1.4-fold slower than that of men [11, 21, 22], which may explain the average longevity of women.

### 6.2. The Ageing Rate Is Not Constant during the Whole Life of Human Beings

According to the previous studies, the ageing rate is not constant through the whole life of human, and it seems to follow an exponential curve. For both men and women, the ageing rate increases slowly before the age of 65 years; then the rates of ageing rapidly advance [21, 22]. Compared with younger people, the rate of the elders is 1.8-fold [21]. The higher ageing rate can explain why there are more diseases and higher death rate in the elders. However, Bai's study showed a little difference, which reported that there is no difference between the ageing rates of people from 65 to 75 years old and people above 75 years old. This had not been reported since other researchers did not give a more detailed division of people over 65 years old [12]. Bai et al. explained this phenomenon as the stability of ageing process over 75 years old.

## 7. Concluding Remarks

With the development of the BA formula, PCA was proposed in BA studies, which may avoid some of the statistical deficiencies of the MLR method [27]. Though the KDM appeared better than PCA method, the relatively simple calculation procedures still allow the PCA method to be preferable in many recent studies [12, 24, 25]. At the same time, since the BA formula is a prediction model, its utility is necessary to be tested. Future studies may be focused on the mortality prediction and the clinical application, which will pull the BA formula changing from theoretical model to practical method. Ideal ageing biomarkers can assure the veracity of the BA equation. At present, although we have developed a large amount of test items for selection of ageing biomarkers, many possible better biomarkers are still waiting to be found, and TRF length is a good start. The future studies may pay more attention to genetic biomarkers in ageing. We believe that studies of constructing BA formula with PCA will become more complete not only in the methodology, but also in the clinical application.

## Figures and Tables

**Table 1:** The basic information of study population.

Researcher	Year	Country	Sample size	Age range	Health standard
Nakamura et al. [17]	1989	Japan	69 males	Average 42.6 ± 9.4 years	Healthy population
Nakamura et al. [18]	1990	Japan	66 females	20–64 years	Healthy population
Nakamura et al. [19]	1996	Japan	221 males	20–85 years	Healthy population
Ueno et al. [20]	2003	Japan	981 females (cross-sectional study) 110 females (longitudinal study)	20–80 years	Healthy population
Nakamura and Miyao [7]	2003	Japan	86 males	31–77 years	Healthy population, including some volunteers with hypertension, hyperlipidemia, and diabetes
Nakamura and Miyao [21]	2007	Japan	86 males	31–78 years	Healthy population, including some volunteers with hypertension, hyperlipidemia, and diabetes
Nakamura and Miyao [22]	2008	Japan	86 males 93 females Males : females = 0.925 : 1	31–77 years	Healthy population, including some volunteers with hypertension, hyperlipidemia, and diabetes
Park et al. [23]	2009	Korea	1588	30–77 years	Healthy population
Bai et al. [12]	2010	China	392 males 460 females Males : females = 0.852 : 1	30–98 years	Healthy population and some volunteers with subclinical state
Jee et al. [11]	2012	Korea	1604 males 760 females Males : females = 2.111 : 1	30–85 years	Healthy population and some volunteers with early state of diseases
Zhang et al. [24]	2014	China	669 males 684 females Males : females = 0.978 : 1	35–91 years	Healthy population
Zhang et al. [25]	2014	China	69 males 70 females Males : females = 0.986 : 1	35–91 years	Healthy population

**Table 2:** General description of test items.

Test items	Researches
Basic information (age, gender, height, body fat, and blood pressure)	[7, 11, 12, 17–25]
Blood routine	[7, 12, 17–25]
Blood chemistry	[7, 12, 17–25]
Urine routine	[7, 12, 20–25]
Pulmonary function	[7, 12, 17–24]
Cardiovascular ultrasound	[12, 24, 25]
Carotid artery ultrasound	[12, 24, 25]
Sexual hormone	[12]
Electrocardiograph	[7, 12, 23, 25]
Chest radiography	[7, 12, 23]
Abdomen ultrasound	[23]
Gastrointestinal endoscopy	[23]
Questionnaire of living habits	[7, 12, 21, 24, 25]
Genetics	[24, 25]

**Table 3:** Biomarkers of ageing.

Organ system	Biomarker	Epidemiologic studies
Cardiovascular system	Pulse pressure	[12, 25]
	Systolic blood pressure	[11, 17–23]
	Heart rate	[17, 18]
	Intima-media thickness	[12, 24, 25]
	Maximum internal diameter of carotid artery	[24, 25]
	End diastolic velocity	[12]
	Mitral valve annulus ventricular septum of the peak velocity of early filling	[24]
	Mitral valve annulus lateral wall of peak velocity of early filling	[12]
	Mitral annulus peak E anterior wall	[25]
	Ratio of peak velocity of early filling to atrial filling	[12]
Respiratory system	*V*_O_2_max_	[11, 23]
	Forced expiratory volume in 1 s	[11, 20–23]
	Forced vital capacity	[17–19]
	Maximal midexpiratory flow rate 75/25	[24]
Nervous system	Trail making test	[24]
	Digital symbol test	[25]
Renal system	Blood urea nitrogen	[17–19, 21–23]
	Cystatin C	[12, 24, 25]
Liver	Serum albumin	[21–23]
	Glutamic oxaloacetic transaminase	[17, 18]
	Glutamic pyruvic transaminase	[19]
	Ratio of albumin to globulin	[20]
	Lactate dehydrogenase	[17–19]
Hematologic system	Erythrocyte sedimentation rate	[23]
	Mean corpuscular hemoglobin	[20]
	Red blood cell count	[22]
	Hematocrit	[21]
	Haemoglobin concentration	[18, 19]
	Fibrinogen	[12]
Metabolism	Glycosylated hemoglobin	[23]
	Glucose	[19, 20]
	Low density cholesterol	[23]
	Atherogenic index	[17, 18]
	Triglyceride	[18]
	Total cholesterol concentration	[19]
Muscle and fat	Grip strength	[11]
	Soft lean mass	[11]
	Waist circumference	[11, 23]
	Percent body fat	[23]
Sensory system	Hearing threshold	[23]
Genetic index	Telomere restriction fragment	[25]

**Table 4:** Correlation coefficients and redundancy coefficients of each research.

Researcher	Year	Correlation coefficient	Redundancy coefficient
Nakamura et al. [17]	1989	0.24	0.5
Ueno et al. [20]	2003	0.25	0.9
Nakamura and Miyao [21]	2007		0.6
Nakamura and Miyao [22]	2008		0.6
Park et al. [23]	2009	0.15	
Bai et al. [12]	2010	0.25	0.4
Jee et al. [11]	2012	0.12	0.75
Zhang et al. [24]	2014	0.40	0.7
Zhang et al. [25]	2014	0.15	0.7

**Table 5:** Comparisons between the multiple linear regression (MLR), the principal component analysis (PCA), Hochschild's method, and the Klemera and Doubal method (KDM) in biological age (BA) estimates.

Methods	Advantages	Disadvantages
MLR	As an initial method of BA estimates, MLR can detect the stabilization and multicollinearity of the empirical data [26].	The distortion of BA at the regression edge is influenced by mathematical factors, and MLR also ignores the discontinuity of the ageing rate during the whole life of individuals [9, 27, 28]. The BA formula established by MLR is probably disserviceable [8].
PCA	PCA selects and transforms the original biomarkers to a reduced and/or transformed new series of uncorrelated variables [8]. PCA avoids the influence of regression edge in MLR [28] and is easy to be operated. This method generates the uncorrelated variables and provides the information of underlying structure of variables [26].	The final step of the computation resembles the MLR method, and some of the statistical deficiencies of MLR cannot be totally avoided [8].
Hochschild's method	Hochschild's method uses the regression for individual biomarkers and evaluates the biomarkers according to their impact on life expectancy [8].	Hochschild's method is not based on mathematical definition of BA, and the construction mechanism is elusive. In particular, the calculation does not correspond to the optimum algorithm [8]. Moreover, a large number of subjects are needed to be measured when Hochschild's method is adopted for a newly developed system [26].
KDM	KDM is a more reliable predictor of mortality and performed better than the chronological age [16]. KDM gives lower errors than other methods, evaluates the precision of BA estimates, and solves the paradox of biomarker selection according to CA [8].	KDM requires complicated calculations [26].
